# The effects of imaging markers on clinical trajectory in cerebral amyloid angiopathy: a longitudinal study in a memory clinic

**DOI:** 10.1186/s13195-023-01161-5

**Published:** 2023-01-12

**Authors:** Hyemin Jang, Min Young Chun, Hee Jin Kim, Duk L. Na, Sang Won Seo

**Affiliations:** 1 Samsung Alzheimer’s Convergence Research Center, Samsung Medical Center, Seoul, South Korea; 2 Departments of Neurology, Samsung Medical Center, Sungkyunkwan University School of Medicine, 81 Irwon-ro, Gangnam-gu, Seoul, 06351 South Korea; 3 Neuroscience Center, Samsung Medical Center, Seoul, South Korea; 4 Department of Health Sciences and Technology, SAIHST, Sungkyunkwan University, Seoul, South Korea; 5 Happymind Clinic, Seoul, South Korea

**Keywords:** Cerebral amyloid angiopathy, Prognosis, Cognition, Amyloid β, Microbleed, Cortical superficial siderosis

## Abstract

**Background:**

We investigated the relevance of various imaging markers for the clinical trajectory of cerebral amyloid angiopathy (CAA) patients in a memory clinic.

**Methods:**

A total of 226 patients with probable CAA were included in this study with a mean follow-up period of 3.5 ± 2.7 years. Although all had more than one follow-up visit, 173 underwent follow-up Mini-Mental Status Examination (MMSE) and Clinical Dementia Rating Sum of Boxes (CDR-SB) ranging from 2 to 15 time points. Among 226, 122 patients underwent amyloid-β (Aβ) PET imaging. The prevalence of intracerebral hemorrhage (ICH) and its imaging predictors was investigated. The effects of CAA imaging markers and Aβ PET positivity on longitudinal cognition based on the MMSE and CDR-SB were evaluated using mixed effects models.

**Results:**

During the follow-up, 10 (4.4%) patients developed ICH: cortical superficial siderosis (cSS; hazard ratio [HR], 6.45) and previous lobar ICH (HR, 4.9), but lobar cerebral microbleeds (CMBs) were not predictors of ICH development. The presence of CMIs (*p* = 0.045) and Aβ positivity (*p* = 0.002) were associated with worse MMSE trajectory in CAA patients. Regarding CDR-SB trajectory, only Aβ positivity was marginally associated with worse longitudinal change (*p* = 0.050).

**Conclusion:**

The results of the present study indicated that various imaging markers in CAA patients have different clinical relevance and predictive values for further clinical courses.

## Introduction

Cerebral amyloid angiopathy (CAA) is characterized by amyloid β (Aβ) deposition in the small meningeal/cortical arteries [1] and clinically diagnosed using characteristic magnetic resonance imaging (MRI) markers such as strictly lobar intracerebral hemorrhage (ICH), lobar cerebral microbleeds (CMBs), and cortical superficial siderosis (cSS). Recently updated clinic-radiological criteria added additional characteristic imaging markers for CAA, which are enlarged perivascular space in the centrum semiovale (CSO-EPVS) and multi-spot patterned white matter hyperintensities (WMH) [2]. Because lobar ICH is the most common symptomatic presentation of CAA, the predictors of ICH development have been investigated in many studies. In particular, cSS has been considered the single most important risk factor for future ICH [3–9]. However, knowledge regarding predictors of clinical and cognitive decline, which are also regarded as important clinical phenotypes of CAA, is limited, especially in a memory clinic [10].

In our previous study, the hemorrhagic markers, lobar CMBs and cSS, were shown specifically associated with cognitive dysfunction and partially mediated by cortical thinning [11]. However, the cross-sectional study design may have limited the clinical implication in terms of prognosis prediction. Recently, we showed that Aβ PET positivity in CAA is associated with worse cognitive trajectory using longitudinal data, emphasizing the clinical implication of Aβ PET positivity [12]. However, we could not consider the effects of the other CAA-specific MRI markers as prognostic markers, such as cortical microinfarcts (CMIs), which have been investigated as another important contributor to cognition in CAA [13].

Therefore, in the present study, how various CAA-specific imaging markers affect the cognitive decline in a probable CAA cohort in a memory clinic was investigated. First, the prevalence of ICH and its imaging predictors was investigated in a memory clinic. In addition, the effects of the MRI markers as well as Aβ PET positivity on cognitive trajectory were investigated. We hypothesized that the presence of cSS, a higher number of CMBs, and CMIs affect cognitive decline regardless of ICH development in CAA patients in a memory clinic.

## Materials and methods

### Study participants

**Fig. 1:**
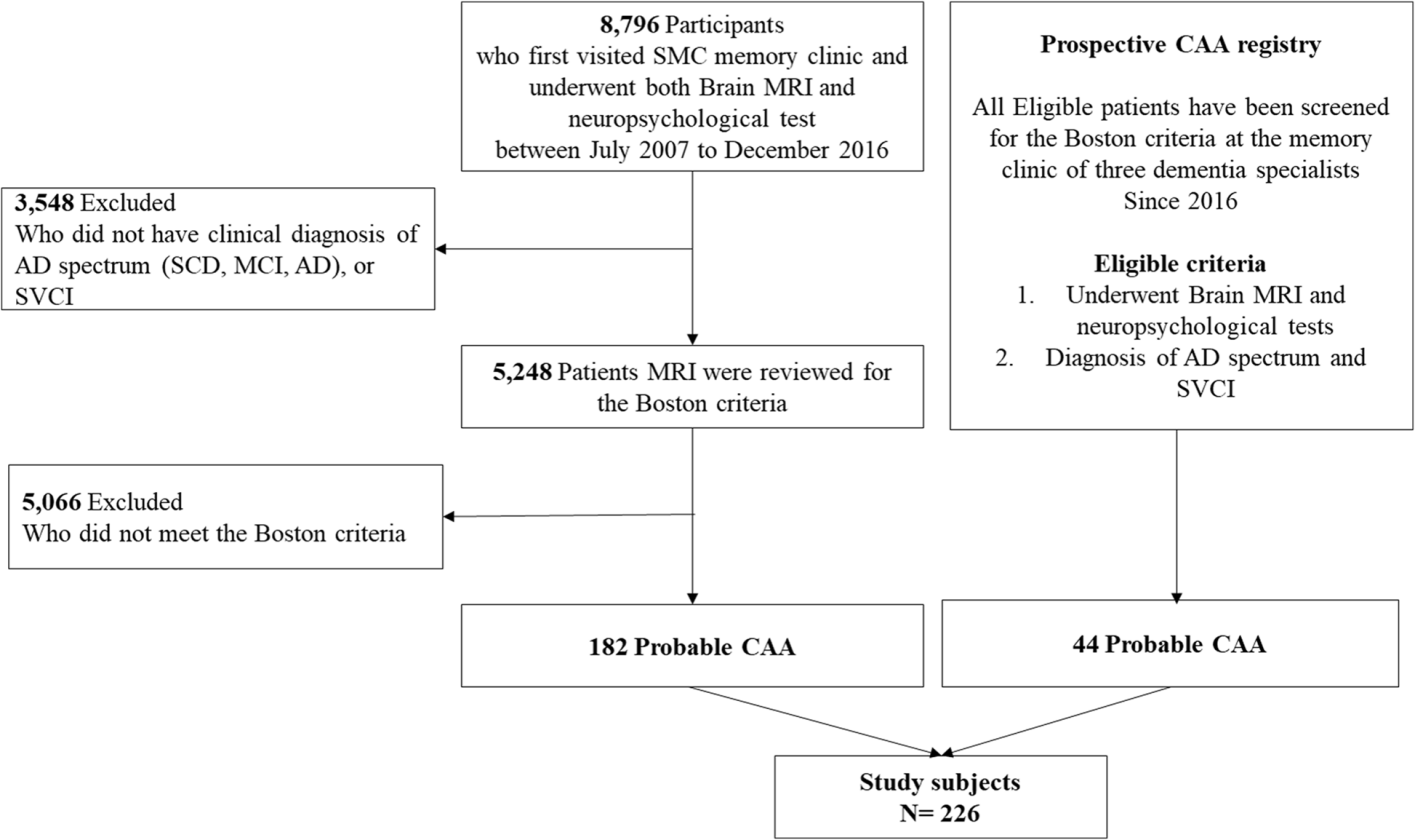
The flowchart of the participant inclusion process. *Abbreviations*: SMC, Samsung medical center; MRI, magnetic resonance images; AD, Alzheimer’s disease; SCD, subjective cognitive decline; MCI, mild cognitive impairment; ADD, Alzheimer’s disease dementia; SVCI, subcortical vascular cognitive impairment; CAA, cerebral amyloid angiopathy

For the selection of study participants, we reviewed Neuroimaging Study Registries of patients who visited a memory clinic in Samsung Medical Center complaining of cognitive impairment. We first reviewed 5248 patients from MRI registries obtained in the memory clinic of Samsung Medical Center between July 2007 to December 2016. Since 2016, we also have developed the CAA imaging registry with compatible patients. Therefore, the present study included a total of 266 probable CAA participants from July 2007 to July 2020. The flowchart detailing the study participant inclusion is shown in Fig. 1. All participants underwent brain MRI at baseline and had at least two strictly lobar ICH/CMBs or one strictly lobar ICH/CMBs with cSS on MRI, meeting both the modified and the recent version 2.0 Boston criteria for CAA [2, 14–16]. All patients underwent baseline Mini-Mental State Examination (MMSE) and completed neuropsychological tests (if their MMSE score was not lower than 10).

### Assessment of cerebral small vessel disease (CSVD) imaging markers on MRI

All participants underwent brain MRI including T2* GRE and FLAIR with or without 3-dimension (3D) T1 images at baseline. Imaging analysis was performed by neurologists who were trained in neuroimaging rating. All structural imaging markers of cerebral small vessel disease (CSVD) were rated in accordance with consensus guidelines [17]. Lobar CMBs were defined as homogenous and round lesions with signal loss (≤ 10 mm in diameter) on T2* GRE images, with a location in exclusively cortical and subcortical areas. Lesions with a diameter > 10 mm were counted as ICH. cSS was defined as linear hypointensities on T2* GRE images, consistent with chronic blood residues on the superficial cortical layers [18]. Four experienced neurologists who were blinded to clinical data rated lobar CMBs and cSS. The interobserver intraclass correlation coefficient (ICC) ranged from 0.87 to 0.91 for lobar CMBs and from 0.82 to 0.96 for cSS [19]. Lacunes were identified and counted in accordance with STandards for ReportIng Vascular changes on nEuroimaging (STRIVE) [17]. The severity of WMHs was rated using the modified Fazekas scale [20]. As the Boston criteria v2.0 introduced CSO-EPVS and WMH in a multi-spot pattern as major imaging characteristics of CAA, we also scored these two imaging markers. We used a validated Visual Rating Scale to score CSO-EPVS on the axial T2-weighted images: 0, no EPVS; 1, < 10 EPVS; 2, 10 to 20 EPVS; 3, 21 to 40 EPVS; and 4, > 40 EPVS [21], and defined severe CSO-EPVS as more than 20 visible EPVS (EPVS grade 3 or 4) according to the Boston criteria v2.0. In each case, the most affected hemisphere was rated, and in cases where the rating was difficult (e.g. due to movement or extensive WMH), we selected the closest category. The multi-spot patterned WMH was defined as the presence of at least 10 small circles or spots of WMH in subcortical areas as described in the previous studies [2, 22]. In addition, we visually rated the number of CMIs using FLAIR, T2-weighted, and T1-weighted images (mostly FLAIR and T1-weighted images). CMIs were defined as hyperintense/hypointense lesions on FLAIR/T1 images, which are less than 5 mm in diameter and restricted to the cortex. CMIs located in regions close to large cortical infarcts and appearing hypointense on GRE (hemorrhagic lesions) were discarded [23–25]. The experienced neurologist (H.J.) underwent ratings for EPVS grade, WMH multi-spot pattern, and CMIs for all images. For randomly selected 20 subjects’ images, an independent neurologist (C.M.Y.) rated all three imaging markers again, and interobserver *κ* were 0.62 for severe CSO-EPVS, 0.88 for a presence of multi-spot patterned WMH, and 0.51 for a presence of CMIs.

### Aβ PET imaging acquisition, preprocessing, and interpretation

Among 226 participants, 122 underwent Aβ PET (26 ^11^C-PiB, 86 ^18^F-florbetaben, 10 ^18^F-flutemetamol PET) using a Discovery STe PET/CT scanner (GE Medical Systems, Milwaukee, WI, USA) in a 3D scanning mode that examined 47 slices 3.3 mm in thickness spanning the entire brain. A 16-slice helical CT (140 keV, 80 mA; 3.75 mm section width) was performed for attenuation correction. For ^11^C-PiB PET, a 30-min emission static PET scan was performed 60 min after injection into an antecubital vein as a bolus of a mean dose of 420 MBq. For ^18^F-florbetaben PET and ^18^F-flutemetamol PET, a 20-min emission PET scan with dynamic mode (consisting of 4 × 5 min frames) was performed 90 min after injection of a mean dose of 311.5 MBq ^18^F-florbetaben and 197.7 MBq ^18^F-flutemetamol, respectively.

For PiB PET, both MR and PET images were co-registered with each other using the rigid body transformation. The T1-weighted MR image of each subject was aligned with the MNI-152 template using a non-linear deformation including translation, rotation, scaling, and shearing. After standard space registration, the grey matter was divided into 116 regions using the automated anatomical labeling (AAL) atlas [26]. To compute the standardized uptake value ratios (SUVRs), every voxel intensity was normalized by the mean intensity of cerebellar gray matter which was regarded as the reference region. Then, global PiB retention ratios were assessed from the volume-weighted average SUVR of 28 bilateral cerebral cortical volumes of interest (VOIs).

Aβ PET positivity was defined when the global PiB SUVR was > 1.5, when florbetaben PET was visually rated as 2 or 3 on the brain Aβ plaque load (BAPL) scoring system [27], and when flutemetamol PET was visually assessed as positive in any 1 of the 5 brain regions (frontal, parietal, posterior cingulate/precuneus, striatum, and lateral temporal lobes) in either hemisphere [28]. Aβ PET images were reviewed by one nuclear medicine physician who was blinded to clinical information and one neurologist. They discussed the discrepant results of Aβ positivity to achieve consensus.

### Neuropsychological tests

All patients underwent neuropsychological tests using the Seoul Neuropsychological Screening Battery (SNSB) [19, 29] which consists of tests for attention, language, visuoconstructive function, verbal and visual memory, and frontal/executive function. In the present study, a summary score was generated for each cognitive domain. An attention score was calculated by summing scores in digit span forward (range 0–9) and digit span backward (range 0–8). A memory domain score (memory score) was calculated by summing scores in verbal and visual memory tests including raw scores on the Seoul Verbal Learning Test (SVLT) immediate recall (range 0–36), delayed recall (range 0–12), and recognition (range 0–24), as well as raw scores on Rey–Osterrieth Complex Figure Test (RCFT) immediate recall (range 0–36), delayed recall (range 0–36), and recognition (range 0–24). A frontal-executive domain score (frontal score) was calculated by summing the scores in a category word generation task (animal), a phonemic word generation task, and the Stroop color-reading test (range 0–120). Raw scores on the Korean version of the Boston Naming Test (KBNT) and RCFT copy test were used to construct the language and visuospatial scores, respectively.

### Follow-up

All participants had follow-up clinic visits at least once, and the mean follow-up period from the baseline MRI was 3.5 ± 2.7 years. Among 226 participants, 173 underwent follow-up MMSE or CDR-SB ranging from 2 to 15 time points.

### Statistical analyses

Univariable logistic regression analysis was performed to determine the predictors associated with ICH development, including baseline age, presence of hypertension and hyperlipidemia, smoking status, anticoagulant use, presence of cSS, APOE2 and APOE4 genotype, number of lobar CMBs, previous lobar ICH (either symptomatic or asymptomatic), presence of severe CSO-EPVS, multi-spot patterned WMH, and severe WMH as independent variables. Independent variables which were significant with *p*-value < 0.2 were subsequently included in the multivariable logistic regression analysis. In addition, to investigate the effects of imaging markers cSS, lobar CMBs, CSO-EPVS, multi-spot patterned WMH, presence of CMIs, and Aβ positivity on longitudinal cognitive changes (MMSE and CDR-SB), linear mixed effects models were performed. In this analysis, lobar CMBs were log-transformed to ln (1 + lobar CMBs) due to skewed distribution. Fixed effects were each imaging marker, time from the baseline (time), age, sex, education years, baseline MMSE or CDR-SB scores, and the two-way interaction term for each imaging marker and time (imaging marker × time). Patients were included as random effects. All statistical analyses were performed with STATA/SE version 15.1. Statistical significance was defined as two-tailed *p* < 0.05.

## Results

### Demographics and clinical characteristics of probable CAA participants

A total of 266 probable CAA patients were included in the present study (Table 1). The mean age of CAA participants was 75.4 ± 7.0 years, and the prevalence of females was 54.4%. Regarding imaging markers, the median number of lobar CMBs was 5 (interquartile range 2–16), and 62 patients (27.4%) had cSS. Among 122 patients who underwent Aβ PET scans, 91 (74.6%) showed Aβ positivity. Among 266 CAA patients, the prevalence of APOE2 and APOE4 carriers was 28 (12.5%) and 98 (43.4%), respectively. The prevalence of lacunes, severe CSO-EPVS, CMIs, and previous ICH on MRI (either symptomatic or asymptomatic) was 37.2% (*n* = 84), 26.9% (*n* = 58), 31% (*n* = 70), and 15.9% (*n* = 36), respectively. In terms of WMH characteristics, the prevalence of multi-spot patterned WMH and severe WMH was 70.4% (*n* = 157) and 22.1% (*n* = 50), respectively.

**Table 1:** Clinical characteristics of probable CAA patients

	Total, *n* = 226
Age (years)	75.4 ± 7.0
Sex (female)	123 (54.4)
Education (years)	10.8 ± 5.3
APOE genotype	
APOE2 carrier	28 (12.5)
APOE4 carrier	98 (43.4)
Aβ PET positivity	91/122 (74.6)
Follow-up period (years)	3.5 ± 2.7
MRI finding	
Number of lobar CMBs	5 (2–16)
Presence of cSS	62 (27.4)
Presence of lacunes	84 (37.2)
Severe CSO-EPVS	58 (26.9)
Presence of lobar ICH	36 (15.9)
Severe WMH	50 (22.1)
WHM in a multi-spot pattern	157 (70.4)
Presence of cortical microinfarcts	70 (31.0)
Vascular risk factors	
Hypertension	112 (49.6)
Diabetes	52 (23.0)
Hyperlipidemia	65 (28.8)
Cardiac disease	17 (7.5)
Stroke	33 (14.6)
Current smoking	41 (18.1)
Anticoagulant use	4 (1.78)
Clinical outcome	
Lobar ICH	10 (4.4)
Stroke	8 (3.5)
Seizure	1 (0.4)
MMSE*	21.2 ± 6.5
CDR-SB	3.8 ± 3.5

Values are expressed as means ± standard deviations, numbers (%), or mean (interquartile range)

*Abbreviations*: *Aβ*, amyloid β; *MRI*, magnetic resonance imaging; *CMBs*, cerebral microbleeds; *cSS*, cortical superficial siderosis; *CAA*, cerebral amyloid angiopathy; AD*,* Alzheimer’s disease dementia; *n*, number; *ICH*, intracerebral hemorrhage; *WMH*, white matter hyperintensity; *MMSE*, Mini-Mental Status Examination; *CDR-SB*, Clinical Dementia Rating Sum of Boxes

*p-value after adjusting for age, sex, and education

### Predictors of lobar ICH development

During the mean follow-up period of 3.5 years, among 226 patients, 10 patients developed lobar ICH, 8 patients presented with ischemic stroke, and 1 patient deteriorated clinically with seizure disorder (Table 1).

In univariate analyses, hypertension (hazard ratio (HR), 0.24; confidence interval (CI), 0.05, 1.16), anticoagulant use (HR, 7.89; CI 0.75–83.5), presence of cSS (HR, 1.02; CI 0.93–1.12), APOE2 (HR, 5.33; CI 1.4–20.25), APOE4 (HR, 0.14; CI 0.02–1.0), and previous lobar ICH (HR, 9.3; CI 2.48– 34.9) were associated with ICH development. However, age, hyperlipidemia, smoking, e4 genotypes, lobar CMBs, severe CSO-EPVS, multi-spot patterned WMH, and severe WMH were not associated with ICH development.

Multivariable analyses including significant predictors from univariate analyses showed that only the presence of cSS (HR, 7.38; CI 1.42–38.44) and previous lobar ICH (HR, 5.07; CI 1.23–20.87) were associated with ICH development but not hypertension, smoking, and APOE genotypes (Table 2).

**Table 2:** Univariable and multivariable logistic regression analysis results for predictors of lobar ICH development

	Univariable		Multivariable	
	HR (CI)^a^	*p*-value	HR (CI)^a^	*p*-value
Age	1.02 (0.93, 1.12)	0.719	NA	
Hypertension	0.24 (0.05, 1.16)	0.076	0.23 (0.04, 1.33)	0.101
Hyperlipidemia	0.27 (0.04, 2.09)	0.211	NA	
Smoking	0.49 (0.06, 3.97)	0.503	NA	
Anticoagulant use	7.89 (0.75, 83.5)	0.086	7.06 (0.18, 272.92)	0.294
Presence of cSS	12 (2.47, 58.25)	0.002	6.45 (1.21, 34.5)	0.029
APOE2	5.33 (1.4, 20.25)	0.014	2.55 (0.52, 12.38)	0.246
APOE4	0.14 (0.02, 1.09)	0.061	0.23 (0.02, 2.29)	0.21
ln(1 + lobar CMBs)	0.97 (0.58, 1.61)	0.911	NA	
Previous lobar ICH	9.3 (2.48, 34.9)	0.001	4.9 (1.09, 21.92)	0.038
Severe CSO-EPVS	0.46 (0.06, 3.73)	0.468	NA	
Multi-spot WMH	1.03 (0.26, 4.09)	0.970	NA	
Severe WMH	0.86 (0.34, 2.17)	0.746	NA	

Independent variables which were significant with *p*-value < 0.2 in the univariable logistic regression analysis were subsequently included in the multivariable logistic regression analysis

*Abbreviations*: *HR*, hazard ratio; *CI*, confidence interval; *NA*, not applicable; *cSS*, cortical superficial siderosis; *CMBs*, cerebral microbleeds; *ICH*, intracerebral hemorrhage, *WMH*, white matter hyperintensity

### Effects of imaging markers on cognitive decline in CAA patients

**Fig. 2:**
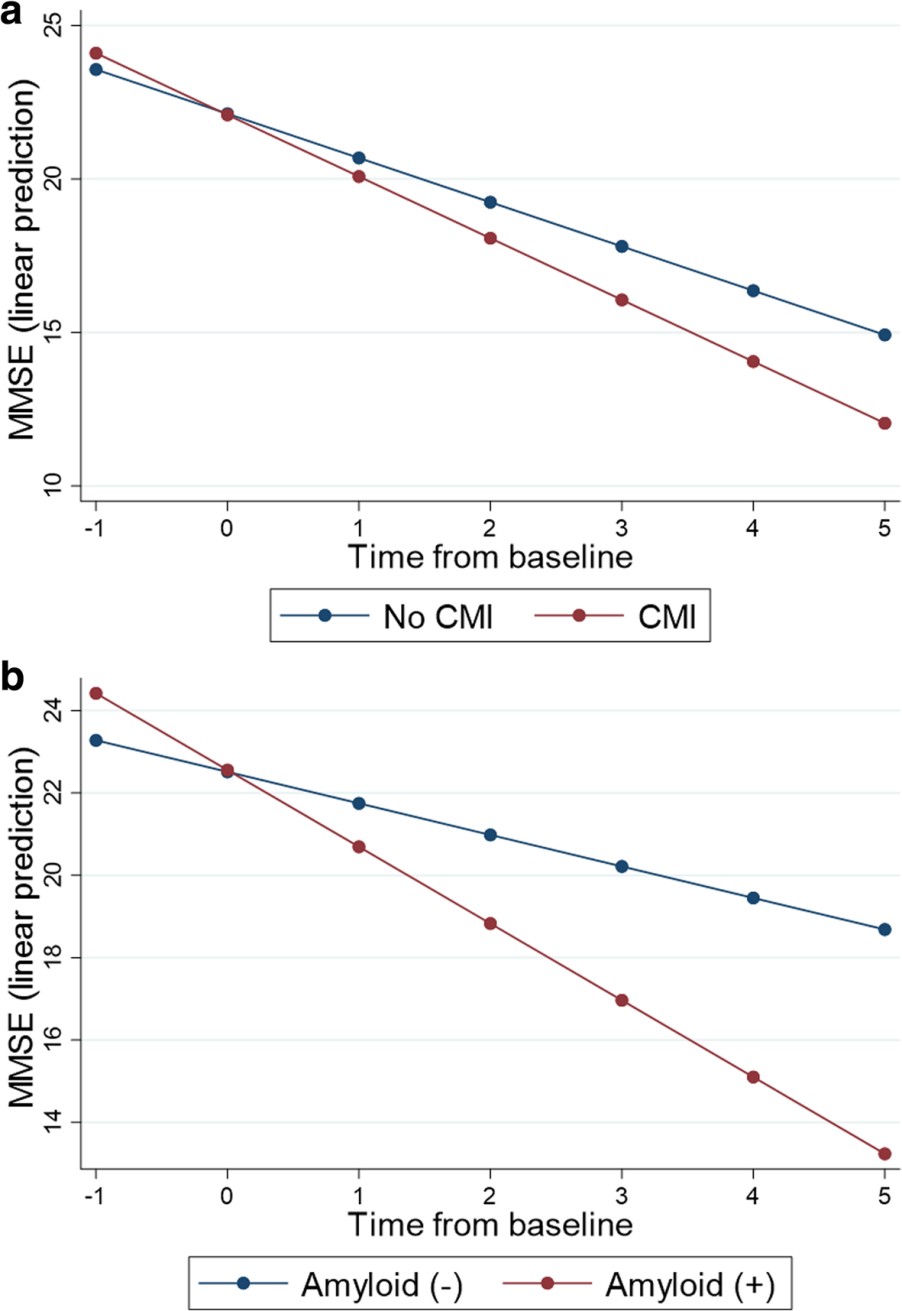
Distinctive MMSE decline based on various imaging markers. **a** CMI. **b** Aβ positivity. *Abbreviations*: MMSE, Mini-Mental State Examination; CMI, cortical microinfarcts; Aβ, amyloid β. *Y*-axis represents the predicted MMSE scores for each follow-up year derived from the predicted model equation using a linear mixed effect model

In linear mixed effects models to investigate the effects of CAA imaging markers on cognitive decline, most of the imaging markers including the presence of cSS, the number of lobar CMBs, severe CSO-EPVS, and multi-spot patterned WMH were not associated with MMSE decline, while only the presence of CMIs (*p* = 0.045) and Aβ positivity (*p* = 0.001) were associated with a decline in MMSE score. However, Aβ positivity (*p* = 0.050) was marginally associated with increased CDR-SB score (Table 3, Fig. 2).

**Table 3:** Effects of imaging markers on longitudinal cognitive trajectory obtained from the mixed effects model

Outcome	Fixed effect	Estimate of fixed effect × time on outcome	
		*B* (SE)	*p*^†^
MMSE		− 0.44 (0.3)	
	Presence of cSS	− 0.14 (0.11)	0.133
	ln(1 + lobar CMBs)	0.04 (0.37)	0.186
	Previous lobar ICH	− 0.57 (0.28)	0.917
	Severe CSO-EPVS	0.01 (0.29)	0.984
	Multi-spot WMH	− 0.48 (0.28)	0.089
	Presence of CMI	− 1.1 (0.36)	0.045
	Aβ positivity	− 0.44 (0.3)	0.002
CDR-SB	Presence of cSS	− 0.44 (0.3)	0.133
	ln(1 + lobar CMBs)	0.04 (0.37)	0.917
	Previous lobar ICH	− 0.34 (0.29)	0.230
	Severe CSO-EPVS	− 0.44 (0.3)	0.440
	Multi-spot WMH	0.04 (0.37)	0.488
	Presence of CMI	− 0.06 (0.23)	0.781
	Aβ positivity	0.55 (0.28)	0.050

*Abbreviations*: *OR*, odds ratio; *CI*, confidence interval; *cSS*, cortical superficial siderosis; *CMBs*, cerebral microbleeds; *ICH*, intracerebral hemorrhage; *CSO-EPVS*, enlarged perivascular space in centrum semiovale; *CMI*, cortical microinfarcts; *WMH*, white matter hyperintensity; *CMI*, cortical microinfarct; *MMSE*, Mini-Mental Status Examination; *CDR-SB*, Clinical Dementia Rating Sum of Boxes; *Aβ*, amyloid β

^†^*P* value (significance) for effects of each imaging marker on longitudinal cognitive changes (= significance for the interaction term of each imaging marker and time interval) obtained from linear mixed effect model

## Discussion

In the present study, clinical characteristics of probable CAA patients based on their CAA-specific MRI markers and their effects on clinical trajectory were investigated using a longitudinal CAA cohort in a memory clinic. The main findings were as follows: First, even in CAA patients from a memory clinic, ICH was an important clinical outcome for which cSS and previous lobar ICH were predictors. Second, only the presence of CMIs and Aβ positivity was associated with the worse trajectory in general cognition in CAA patients. Taken together, the findings indicated that various imaging markers in CAA patients have different clinical relevance and predictive values for further clinical course.

The first major finding was that 4.4% of probable CAA patients in the memory clinic developed ICH, and 3.5% developed stroke during the mean follow-up period of 3.5 years. This prevalence is lower than that in previous reports [5, 8, 30]. This discrepancy may be explained by participants from different CAA cohorts (primarily patients with a relatively low prevalence of previous ICH or cSS recruited from a memory clinic in the present sample versus patients with symptomatic lobar ICH in previous studies), considering that CAA with and without lobar ICH might have different pathophysiologic mechanisms [31]. In the univariate analyses, we found hypertension, anticoagulant use, APOE genotype, presence of cSS, and previous lobar ICH were associated with lobar ICH development (*p* < 0.2). However, in the final model, we found that only cSS and previous ICH were predictors for further ICH development in memory clinic settings, which is consistent with previous studies [3–9, 32]. However, the number of lobar CMBs was not a significant predictor of ICH in the present study. In several previous studies, two different imaging phenotypes in CAA were suggested, macrohemorrhagic (cSS and ICH) and microhemorrhagic (many lobar CMBs) types [33–35]. Although the negative association of the lobar CMBs and ICH development in this study might be due to the lack of power, it might alternatively indicate that the presence of lobar CMBs and cSS/ICH in a memory clinic cohort may not be potentially mutually associated and occur simultaneously. Thus, in patients who have many lobar CMBs but no cSS or previous ICH, the need for a reliable indicator of future ICH should be further studied.

Second, only the presence of CMIs was associated with general cognition decline represented by MMSE score among various imaging markers on MRI. CMIs are found commonly in the brains with AD and CAA pathologies, known to be associated with CAA severity [36, 37]. As an important imaging marker in CAA, we found that 31% of study participants had CMIs, which is similar with the prevalence reported in the previous CAA cohort [24]. Our finding that the presence of CMIs was associated with MMSE decline corroborates the previous knowledge that CMIs are important in cognition [24, 38]. However, in this study, we failed to demonstrate the association between the other imaging markers (particularly, the presence of cSS and the number of lobar CMBs) and cognitive trajectory. In terms of the association between hemorrhagic markers and longitudinal clinical follow-up, cSS was mainly studied as the key predictive marker for future ICH [3–9]. Our study finding suggests that although cSS is strongly associated with ICH occurrence, it does not necessarily cause a detrimental effect on cognition. In terms of CMBs, several longitudinal studies as well as cross-sectional studies investigated the association between lobar CMBs and cognitive deterioration [11, 39–43], but most of these studies were population-based studies which could not reveal the dose-effect relationship between lobar CMBs and cognitive change specifically in probable CAA patients, which our current study tried to investigate. Therefore, we consider that in probable CAA patients in a memory clinic, the number of lobar CMBs might not be directly associated with cognitive decline.

Our finding demonstrated that Aβ positivity had the single most important effect on cognitive decline (presented by MMSE and marginally by CDR-SB) in CAA in a memory clinic setting. First of all, the prevalence of Aβ positivity (74.6%) in this study was similar to those of 60% [44] or 70 %[45] in previous studies, although other studies showed relatively high sensitivity over 80% of Aβ PET in probable CAA [46–49]. The clinical significance of Aβ positivity in CAA is consistent with our previous study showing that Aβ positivity was associated with cognitive decline in CAA patients, although the effects of other hemorrhagic markers were not previously considered [12]. As discussed in the previous study, Aβ positivity in CAA patients may indicate combined AD neuropathological changes or advanced CAA pathology even without parenchymal Aβ [12]. Thus, the synergistic effect of combined AD or worse vascular injury due to severe CAA may contribute to cognitive decline [50, 51]. However, in this study, not all patients included did not undergo Aβ PET, which limits the relevance of the study finding. Therefore, a future study with a more complete dataset is required.

## Limitation

The present study’s strengths include a relatively large probable CAA cohort from a memory clinic compared with previous studies [44–49]. Although our findings on the predictors of lobar ICH development or cognitive decline in CAA might be predictable based on many previous studies, our study has generated additional evidence supporting the previous knowledge, especially using the real memory clinic cohort. However, several limitations should be acknowledged. First, this study was not confirmed by pathological data. This could be a critical limitation of this study, because the modified Boston criteria (v1.5) have not been fully validated in a memory clinic. Second, the retrospectively recruited cohort could have led to underestimated ICH occurrence or cognitive decline because patients with rapid deterioration might have been lost to follow-up. Third, there might be possibilities that some potential CAA patients were not included because v1.5 is less sensitive than the recent version (v2.0) of the Boston criteria and if individual specialists missed the CAA cases in the clinic while screening for probable CAA. Fourth, a few patients developed ICH or stroke during the follow-up period, where negative associations with the presence of lobar CMBs or Aβ positivity on PET simply might be simply due to a lack of power. Finally, Aβ PET was not performed in all participants which may limit the study findings.

## Conclusions

The results of the present study are noteworthy because the effects of CAA-related imaging markers on longitudinal cognition in memory clinics were reported for the first time. The results indicated that cSS and previous lobar ICH were associated with lobar ICH development, while CMIs and Aβ positivity which indicates AD pathology in the parenchyma were associated with cognitive decline in a memory clinic setting.

## Data Availability

The data are publicly available and provided upon request.
